# Physical activity and risk of adverse events in atrial fibrillation: evidence from European and Asian cohorts

**DOI:** 10.1093/europace/euag032

**Published:** 2026-03-01

**Authors:** Michele Rossi, Tommaso Bucci, Enrico Tartaglia, Amir Askarinejad, Steven Ho Man Lam, Andrea Galeazzo Rigutini, Claudio Ferri, Giuseppe Boriani, Hung-Fat Tse, Tze-Fan Chao, Gregory Y H Lip

**Affiliations:** Liverpool Centre for Cardiovascular Science at University of Liverpool, Liverpool John Moores University and Liverpool Heart and Chest Hospital, 6 West Derby Street, Liverpool L7 8TX, UK; Department of Life, Health & Environmental Sciences, University of L’Aquila, L’Aquila, Italy; Internal Medicine and Nephrology Division, ASL1 Avezzano-Sulmona-L’Aquila, San Salvatore Hospital, L’Aquila, Italy; Liverpool Centre for Cardiovascular Science at University of Liverpool, Liverpool John Moores University and Liverpool Heart and Chest Hospital, 6 West Derby Street, Liverpool L7 8TX, UK; Internal, Vascular and Emergency Medicine – Stroke Unit, University of Perugia, Perugia, Italy; Liverpool Centre for Cardiovascular Science at University of Liverpool, Liverpool John Moores University and Liverpool Heart and Chest Hospital, 6 West Derby Street, Liverpool L7 8TX, UK; Division, Department of Biomedical, Metabolic and Neural Sciences, University of Modena and Reggio Emilia, Policlinico di Modena, Modena, Italy; Liverpool Centre for Cardiovascular Science at University of Liverpool, Liverpool John Moores University and Liverpool Heart and Chest Hospital, 6 West Derby Street, Liverpool L7 8TX, UK; Liverpool Centre for Cardiovascular Science at University of Liverpool, Liverpool John Moores University and Liverpool Heart and Chest Hospital, 6 West Derby Street, Liverpool L7 8TX, UK; Liverpool Centre for Cardiovascular Science at University of Liverpool, Liverpool John Moores University and Liverpool Heart and Chest Hospital, 6 West Derby Street, Liverpool L7 8TX, UK; Internal, Vascular and Emergency Medicine – Stroke Unit, University of Perugia, Perugia, Italy; Department of Life, Health & Environmental Sciences, University of L’Aquila, L’Aquila, Italy; Internal Medicine and Nephrology Division, ASL1 Avezzano-Sulmona-L’Aquila, San Salvatore Hospital, L’Aquila, Italy; Division, Department of Biomedical, Metabolic and Neural Sciences, University of Modena and Reggio Emilia, Policlinico di Modena, Modena, Italy; Division of Cardiology, Department of Medicine, School of Clinical Medicine; Queen Mary Hospital, The University of Hong Kong, Hong Kong SAR, China; Division of Cardiology, Department of Medicine, Taipei Veterans General Hospital, NO. 201, Sec. 2, Shipai Rd., Beitou District, Taipei 112201, Taiwan; Institute of Clinical Medicine and Cardiovascular Research Center, National Yang Ming Chiao Tung University, Taipei, Taiwan; Liverpool Centre for Cardiovascular Science at University of Liverpool, Liverpool John Moores University and Liverpool Heart and Chest Hospital, 6 West Derby Street, Liverpool L7 8TX, UK; Danish Center for Health Services Research, Department of Clinical Medicine, Aalborg University, Aalborg, Denmark; Department of Cardiology, Lipidology and Internal Medicine, Medical University of Bialystok, Bialystok, Poland

**Keywords:** Atrial fibrillation, Physical activity, Cardiovascular outcomes, Mortality, ABC pathway

## Abstract

**Aims:**

To evaluate differences in clinical characteristics and outcomes based on physical activity levels in patients with atrial fibrillation (AF), comparing Europeans and Asians.

**Methods and results:**

Post-hoc analysis of two prospective registries from Europe and the Asia-Pacific. Patients were classified as *inactive* (no exercise or <3 h/week) or *active* (≥3 h/week). The primary outcome was a composite of all-cause death and major adverse cardiovascular events (MACE). Secondary outcomes included all-cause death, MACE, major bleeding, individual MACE components. Cox model estimated hazard ratios (HRs) and 95% confidence intervals (CIs) for outcomes. Subgroup analyses were performed by clinically relevant variables and enrolment setting. Of 13 126 participants (69 ± 12 years; 39% female), 3639 (28%) were *physically active* and 9487 (72%) *physically inactive*. Across both groups, Asians had lower odds of obesity, symptomatic AF and heart failure, but higher odds of cardiovascular risk factors than Europeans. After a median follow-up of 514 days, *physically active* AF patients had a lower risk of composite outcome (HR 0.66, 95%CI 0.56–0.78), all-cause death (HR 0.52, 95%CI 0.42–0.65), MACE (HR 0.80, 95%CI 0.65–0.99), cardiovascular death (HR 0.60, 95%CI 0.42–0.86), with no significant differences between Europeans and Asians (*p*_interaction_ for composite outcome = 0.298). The risk of the composite outcome decreased progressively with increasing levels of physical activity, with no significant differences between Europeans and Asians (*p*_interaction_ = 0.845).

**Conclusion:**

In patients with AF, self-reported physical activity is associated with a lower risk of adverse events, consistently across Europe and Asia. Physical activity may represent a component of a lower-risk clinical profile in AF.

## Graphical Abstract

**Graphical Abstract:**
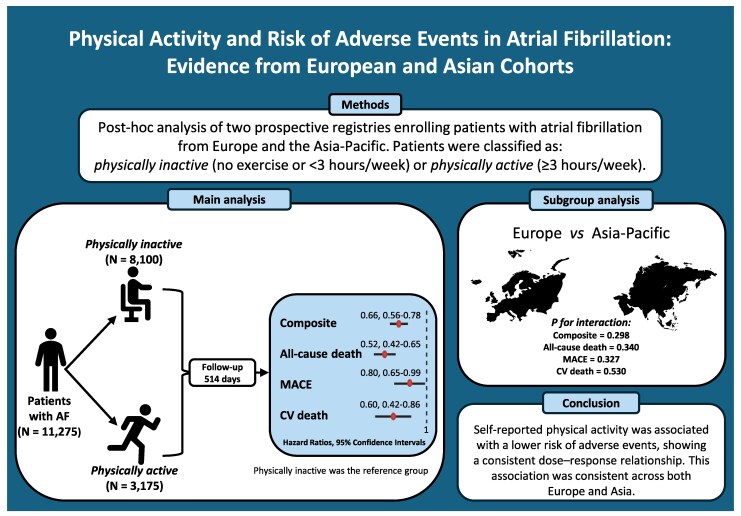

What’s new?

- In a large contemporary cohort of patients with atrial fibrillation from Europe and Asia, self-reported physical activity was associated with a lower risk of adverse clinical outcomes.
- The association was consistent across geographically and clinically distinct populations.
- Physical activity may identify a lower-risk clinical phenotype across different healthcare settings.

## Introduction

Physical inactivity is associated with an increased risk of cardiovascular events and overall mortality.^1^ Globally, nearly one-third of adults (31.3%, 1.8 billion) were classified as insufficiently physically active in 2022, with the highest prevalence observed in the high-income Asia-Pacific region, where rates have continued to rise over recent years.^2^ In contrast, high-income Western countries have recently shown a trend towards reduced levels of physical inactivity.^2^

Atrial fibrillation (AF), the most common cardiac arrhythmia worldwide, is associated with an increased risk of cardiovascular morbidity and mortality.^3^ Several studies have shown that physical activity is linked to a reduced risk of developing AF, even amongst patients with risk factors for its development.^4–7^ Cohort studies in the general population and patients with AF have shown that higher levels of regular physical activity are associated with reduced risk of all-cause and cardiovascular mortality, sudden cardiac death, heart failure and dementia, although the evidence for stroke prevention remains inconclusive.^8–10^ This is particularly relevant given that individuals with AF may engage in less daily physical activity compared to those without AF, and that greater AF symptom severity may be associated with reduced levels of exercise.^11^

This study aimed to assess differences in clinical characteristics and outcomes between physically active and physically inactive patients with AF, using data from two large prospective registries in Europe and Asia, with a particular focus on regional variations between the two enrolment settings.

## Methods

Data were obtained from two prospective, observational registries comprising patients with AF across Europe and East Asia. The EURObservational Research Programme (EORP) AF Long-Term General Registry, initiated by the European Society of Cardiology (ESC), was a large-scale, multicentre, observational study that enrolled patients with AF attending routine cardiology care in 250 centres across 27 European countries.^12^ Recruitment occurred consecutively from October 2013 to September 2016, including individuals diagnosed with AF either as a primary or secondary diagnosis of AF during inpatient or outpatient cardiology consultations. The participating sites were distributed throughout Europe, ensuring broad geographic and demographic representation of the continental AF population. Study approval was obtained from the relevant national authorities and ethics committees at each site, and the registry was conducted in accordance with the EU’s Good Clinical Practice guidelines (CPMP/ECH/135/95) and the Declaration of Helsinki.^12^

The Asian Pacific Heart Rhythm Society (APHRS) registry^13^ adopted the EORP-AF protocol, enrolling consecutive patients diagnosed with AF at cardiology clinics in tertiary and general hospitals across five Asia-Pacific regions: Hong Kong, South Korea, Japan, Singapore, and Taiwan. Patient inclusion began in 2015 and concluded in 2017. Ethical approval was secured from local review boards, and the registry was registered on ClinicalTrials.gov (NCT04807049).

Baseline data collection included demographics, clinical history, and ongoing pharmacological therapy, all recorded via a harmonized electronic case report form (eCRF) that was uniformly applied across both registries. According to the respective protocols, patients enrolled in the EORP registry were followed up for two years, while those in the APHRS registry were observed over a one-year period. Major clinical events were documented throughout the follow-up duration.

### Physically inactive and physically active definitions

At the time of enrolment, patients were asked to self-report their physical activity levels, which were categorized as follows: none (no exercise or < 3 h/week for < 2 years), occasional (< 3 h/week for ≥ 2 years), regular (≥ 3 h/week for ≥ 2 years), and intense (> 7 h/week for ≥ 2 years). The reported hours per week referred to any type of physical activity, without a formal distinction between moderate- and vigorous-intensity activity. Based on the 2020 WHO physical activity guidelines that recommend adults do at least 150 min of moderate-intensity activity per week,^14^ the ≥3 h/week threshold was selected as a pragmatic and conservative approximation of guideline-recommended physical activity within the constraints of the available categorical data. Accordingly, we divided the population into two groups: a *physically inactive* AF group, which included individuals reporting ‘none’ or ‘occasional’ activity, and a *physically active* AF group, which included those reporting ‘regular’ or ‘intense’ activity.

### Symptom controlled definition

Patients were classified into two groups based on their baseline EHRA score: symptom-controlled AF (EHRA I or II) and symptomatic AF (EHRA III or IV).

### Clinical scores

Thromboembolic risk was assessed using the CHA_2_DS_2_-VASc and CHA_2_DS_2_-VA scores,^15–17^ whereas bleeding risk was assessed with the HAS-BLED score.^18^ In accordance with the guidelines available at the time of registry enrolment,^19,20^ oral anticoagulation (OAC) was deemed appropriate for male patients with a CHA_2_ DS_2_-VASc score ≥1 and for female patients with a score ≥2.

### Adherence to the atrial fibrillation better care pathway

Adherence to the Atrial fibrillation Better Care (ABC) pathway was assessed according to a previously published definition from the ESC–EHRA EORP-AF General Long-Term Registry, based on appropriate oral anticoagulation according to the CHA₂DS₂-VASc score, symptom control (EHRA I–II), and guideline-directed management of major cardiovascular comorbidities.^21^ Patients were classified as ABC-adherent if all three criteria were fulfilled, whereas those with at least one unmet criterion were considered ABC-nonadherent.

### Outcomes

The primary outcome was a composite of all-cause death and major adverse cardiovascular events (MACE, including thromboembolic events, cardiovascular death, and acute coronary syndromes). Secondary outcomes were all-cause death, MACE, and major bleeding. Exploratory analyses were performed to assess the risk of each MACE component.

### Statistical analysis

Continuous variables were reported as mean ± standard deviation (SD) and compared using Student’s *t*-test. Categorical variables were presented as frequencies and percentages and analysed using the χ² test.

Univariable and multivariable logistic regression analyses were performed to identify factors associated with *physically active* AF and were expressed as Odds Ratios (OR) with their 95% Confidence Intervals (CI). Multivariable model was adjusted for the following variables: age ≥ 75 years, sex, enrolment setting (APHRS vs. EORP), obesity, symptom control, hypertension, diabetes mellitus, coronary artery disease (CAD), peripheral vascular disease (PAD), heart failure, previous thromboembolic events, chronic kidney disease (CKD), dementia, anaemia and use of oral anticoagulants (OACs). Obesity was defined as a Body Mass Index (BMI) ≥ 30 kg/m². Similar multivariable logistic models were performed separately to identify factors associated with being from the Asian group among physically active and physically inactive patients, excluding enrolment setting as a covariate.

Annual incidence rates of adverse outcomes were calculated as the number of first occurrences of events per total person-years, expressed per 100 person-years, with a corresponding 95%CI. Comparisons of annual incidence rates between groups were performed using the Poisson test. Differences in cumulative risk for the composite outcome between *physically active* AF and *physically inactive* AF were assessed using the log-rank test and represented through Kaplan–Meier curves.

Univariable and multivariable Cox proportional hazards regression analyses were used to calculate the hazard ratios (HRs) and 95%CI for the risk of adverse events. The *physically inactive* AF group was used as the reference category. All Cox multivariable models were adjusted for the same variables included in the multivariable logistic regression model.

To assess whether the proportional hazards assumption held in the Cox regression model for the risk of primary outcome, we applied a *χ*^2^ test based on Schoenfeld residuals.

In addition to the primary adjusted analysis, several sensitivity analyses were performed to assess the robustness of the findings. First, we examined the risk of adverse events in patients with AF according to different levels of physical activity. Patients were categorized as follows: *no physically active* AF (no exercise or < 3 h/week for < 2 years), *occasional physically active* AF (< 3 h/week for ≥ 2 years), *mild physically active* AF (≥ 3 h/week for ≥ 2 years) and *intense physically active* AF (> 7 h/week for ≥ 2 years).

Second, the risk of the composite outcome was compared between *physically active* and *physically inactive* patients after propensity score matching (PSM). The propensity score was estimated using a logistic regression model, including the same covariates as in the multivariable models. Patients were matched 1:1 using nearest-neighbour matching without replacement, with a calliper of 0.2 on the logit scale. Covariate balance was assessed using standardized mean differences, with values <0.1 indicating adequate balance. Univariable and multivariable Cox regression models were then applied to the matched cohort to estimate the association between physical activity status and the composite outcome. The proportional hazards assumption for this model was assessed using a *χ*^2^ test based on Schoenfeld residuals.

Third, to assess robustness to unmeasured confounding, *E*-values were calculated for the hazard ratios of physical activity derived from the primary adjusted Cox model and the propensity score–matched analysis.

Fourth, to account for potential differences in anticoagulation strategies, an additional sensitivity analysis was performed by refitting the primary Cox model, replacing overall OACs use (yes/no) with anticoagulant type (no OACs, vitamin K antagonist, or NOACs).

Fifth, potential geographic heterogeneity was explored through interaction analyses by enrolment setting (Europe vs. Asia), as well as across clinically relevant subgroups based on the variables included in the multivariable models.

Finally, given the presence of competing risks in this high-risk population of patients with AF, Fine–Gray subdistribution hazard models were used to account for the competing risk of non-cardiovascular death when evaluating both cardiovascular death, and MACE. Subdistribution hazard ratios (sHRs) with 95% confidence intervals were reported. The multivariable covariates included in the Fine–Gray models were the same as those used in the corresponding multivariable Cox regression analyses.

All statistical analyses were performed with R version 4.3.1 (R Core Team 2020, Vienna, Austria), and a two-sided *P* < 0.05 was considered statistically significant.

## Results

Of the 15 762 AF patients enrolled in the two registries, 13 126 (83%) with available data on physical activity were included in the baseline analysis. Overall, *physically active* AF patients (*n* = 3639; age 68 ± 12 years, 30% female) were younger, more often male, of Asian origin, and had a lower prevalence of obesity, symptomatic AF, and cardiovascular diseases compared to *physically inactive* AF patients (*Table 1*). Moreover, *physically active* AF had lower mean CHA_2_DS_2_-VASc score (2.64 ± 1.65 vs. 3.23 ± 1.79, respectively, *P* < 0.001) and HAS-BLED score (1.45 ± 1.03 vs. 1.59 ± 1.10, respectively *P* < 0.001) but no differences were found in OAC prescription between the two groups. Overall, *physically active* patients showed a higher prevalence of adherence to the ABC pathway (34% vs. 28%, *P* < 0.001, *Table 1*).

**Table 1:** Baseline characteristics of physically inactive and physically active patients with atrial fibrillation.

Characteristic	Physically inactive *n* = 9487	Physically active *n* = 3639	*P*-value
Age	70 ± 12	68 ± 12	<0.001
Sex			<0.001
Male	5423 (57%)	2553 (70%)	
Female	4064 (43%)	1086 (30%)	
Group			<0.001
European	7183 (76%)	2342 (64%)	
Asian	2304 (24%)	1297 (36%)	
BMI	27.7 ± 5.3	26.7 ± 4.5	<0.001
Obesity	2450 (28%)	704 (21%)	<0.001
Smoke	835 (9.1%)	329 (9.2%)	0.8
Symptomatic AF	1669 (18%)	415 (11%)	<0.001
Systolic	132 ± 20	131 ± 19	0.4
Diastolic	78 ± 12	78 ± 12	0.9
Heart rate	82 ± 23	80 ± 21	<0.001
Hypertension	5965 (63%)	2153 (59%)	<0.001
Diabetes	2432 (26%)	678 (19%)	<0.001
Dyslipidaemia	3857 (42%)	1372 (39%)	0.004
Heart failure	3767 (40%)	943 (26%)	<0.001
CKD	1254 (13%)	241 (6.6%)	<0.001
Dementia	178 (1.9%)	33 (0.9%)	<0.001
CAD	2567 (29%)	798 (23%)	<0.001
PAD	668 (7.2%)	148 (4.1%)	<0.001
Pulmonary hypertension	684 (7.3%)	189 (5.2%)	<0.001
Cancer	200 (2.1%)	75 (2.1%)	>0.9
Thromboembolic events	1159 (12%)	382 (11%)	0.005
Ischaemic stroke	649 (6.9%)	219 (6.0%)	0.005
Haemorrhagic event	72 (0.8%)	15 (0.4%)	0.028
Anaemia	670 (7.1%)	164 (4.5%)	<0.001
CHA_2_DS_2_VASc	3.23 ± 1.79	2.64 ± 1.65	<0.001
CHA_2_DS_2_VA	2.80 ± 1.67	2.34 ± 1.56	<0.001
HASBLED	1.59 ± 1.10	1.45 ± 1.03	<0.001
VKAs	4291 (45%)	1327 (36%)	<0.001
OACs	7950 (84%)	3047 (84%)	>0.9
NOACs	3665 (39%)	1720 (47%)	<0.001
Beta blockers	4976 (64%)	1968 (61%)	0.005
ACEin	3472 (37%)	1139 (31%)	<0.001
ARBs	1983 (21%)	745 (21%)	0.7
Statin	4053 (43%)	1445 (40%)	0.003
Insulin	537 (5.7%)	105 (2.9%)	<0.001
Oral antidiabetics	1603 (17%)	442 (12%)	<0.001
Criteria A	7753 (82%)	2970 (82%)	0.9
Criteria B	7817 (82%)	3224 (89%)	<0.001
Criteria C	3757 (42%)	1751 (50%)	<0.001
ABC adherent	2505 (28%)	1197 (34%)	<0.001

BMI, body mass index; CKD, chronic kidney disease; CAD, coronary artery disease; PAD, peripheral vascular disease; NOAC, non-vitamin K antagonist anticoagulant; OACs, oral anti-coagulants; ACE-I, angiotensin converting enzyme inhibitors; ARBs, angiotensin receptor blockers, ABC, Atrial fibrillation Better Care pathway.

Multivariable logistic regression analysis identified that *physically active* AF patients were less likely to be aged ≥ 75 years (OR 0.84, 95%CI 0.77–0.93), female (OR 0.57, 95%CI 0.52–0.63), obese (OR 0.74, 95%CI 0.67–0.82), or to have symptomatic AF (OR 0.78, 95%CI 0.67–0.89). Additionally, *physically active* AF individuals exhibited lower odds of cardiovascular comorbidities, including diabetes (OR 0.78, 95%CI 0.70–0.87), heart failure (OR 0.65, 95%CI 0.59–0.72), CKD (OR 0.59, 95%CI 0.50–0.70), CAD (OR 0.89, 95%CI 0.81–1.00) and PAD (OR 0.80, 95%CI 0.65–0.98). *Physically active* AF patients were also more likely to be of Asian origin (OR 1.41, 95% CI 1.28–1.55). No significant associations were observed with hypertension, thromboembolic events, anaemia, or OAC prescription (see Supplementary material online, *Table S1*).

### European vs. Asian patients

Among the 3639 *physically active* AF patients, 1297 (36%) were from the APHRS-AF Registry and 2342 (64%) from the EORP-AF Registry. Asian patients (age 71 ± 11 years, 32% female) were older, exhibited a higher prevalence of cardiovascular comorbidities, but a lower prevalence of obesity, symptomatic AF and heart failure compared to their European counterparts (see Supplementary material online, *Table S2*).

Among the 9487 *physically inactive* AF patients, 2304 (24%) were from the APHRS-AF Registry and 7138 (76%) from the EORP-AF Registry. Asian patients (age 68 ± 12 years, 36% female) were younger, more often male, and had a lower prevalence of symptomatic AF, cardiovascular comorbidities and heart failure compared to their European counterparts (see Supplementary material online, *Table S3*).

Multivariable logistic regression analysis identified that *physically active* Asian patients were less likely to be obese (OR 0.32, 95%CI 0.26–0.40) and had lower odds of symptomatic AF (OR 0.30, 95%CI 0.21–0.40), and heart failure (OR 0.72, 95%CI 0.60–0.88); however, they exhibited higher odds of hypertension (OR 1.54, 95%CI 1.31–1.82), and diabetes (OR 1.99, 95%CI 1.63–2.44, *Table 2*).

**Table 2:** Multivariable logistic regression analysis of factors associated with belonging to the Asian group among *physically active* and *physically inactive* patients with atrial fibrillation

Characteristic	Physically active OR (95% CI)	Physically inactive OR (95% CI)
Age ≥ 75	1.27 (1.06–1.51)*	0.70 (0.62–0.79)*
Female	1.05 (0.88–1.24)	0.73 (0.66–0.83)*
Obesity	0.32 (0.26–0.40)*	0.28 (0.24–0.33)*
Symptomatic AF	0.30 (0.21–0.40)*	0.37 (0.30–0.44)*
Hypertension	1.54 (1.31–1.82)*	1.13 (1.00–1.27)*
Diabetes	1.99 (1.63–2.44)*	1.40 (1.23–1.60)*
Heart failure	0.72 (0.60–0.88)*	0.46 (0.41–0.53)*
CKD	0.83 (0.58–1.16)	0.77 (0.63–0.93)*
Dementia	2.03 (0.79–5.56)	1.53 (0.96–2.37)
Thromboembolic events	1.51 (1.18–1.95)*	1.16 (0.97–1.38)
CAD	0.86 (0.71–1.04)	0.69 (0.61–0.79)*
PAD	0.14 (0.07–0.26)*	0.13 (0.08–0.20)*
Anaemia	3.90 (2.59–5.96)*	1.77 (1.41–2.23)*
OACs	0.78 (0.63–0.97)*	0.83 (0.71–0.96)*

The European group was used as the reference.

AF, hypertension; CAD, coronary artery disease; PAD, peripheral vascular disease; CKD, chronic kidney disease; OACs, oral anticoagulants.

Asterisks indicate statistical significance at *P* < 0.05.

Among *physically inactive* patients, multivariable logistic regression analysis identified that Asian individuals were less likely to be female (OR 0.73, 95%CI 0.66–0.83), obese (OR 0.28, 95%CI 0.24–0.33), and had lower odds of symptomatic AF (OR 0.37, 95%CI 0.30–0.44), and heart failure (OR 0.46, 95%CI 0.41–0.53); however, they exhibited higher odds of hypertension (OR 1.13 95%CI 1.00–1.27), and diabetes (OR 1.40, 95%CI 1.23–1.60, *Table 2*).

### Survival analysis

Of the initial population, incomplete follow-up data led to the exclusion of 464 (13%) patients from the *physically active* AF group and 1387 (15%) patients from the *physically inactive* AF group (see Supplementary material online, *Figure S1*). Patients excluded were more likely to be from the APHRS-AF Registry, smokers, affected by CAD, but less likely to be obese and receiving OAC compared to those included. No significant differences were observed in terms of age, sex, other cardiovascular comorbidities, CHA₂DS₂-VASc/CHA₂DS₂-VA or HAS-BLED scores (see Supplementary material online, *Table S4*). The final population included in the survival analysis comprised 11 275 patients (86% of the initial cohort), of whom 3175 (28%) belonged to the *physically active* AF group and 8100 (72%) to the *physically inactive* AF group (see Supplementary material online, *Figure S1*).

After a median follow-up of 514 days (IQR: 263–768), the following adverse events were recorded: 1514 (13.4%) composite outcome, 993 (8.8%) all-cause death, 953 (8.5%) MACE, 372 (3.3%) cardiovascular death, 418 (3.7%) acute coronary syndrome, 230 (2.0%) thromboembolic events, and 235 (2.1%) major bleeding. *Physically active* AF patients experienced significantly lower annual incidence rates of composite outcome, all-cause death, MACE, cardiovascular death, and acute coronary syndromes compared to *physically inactive* AF patients (*Table 3*). No significant differences were observed in terms of incidence rates for the other outcomes.

**Table 3:** Number of adverse events, incidence rates, and multivariable cox regression analysis for primary and secondary outcomes in physically active vs. physically inactive patients with atrial fibrillation

	Number of events	Incidence rate per 100 patient-years (95%CI)	*P*-value	Multivariable HR (95%CI)
**Composite outcome**				
Physically active AF	240	4.71 (4.13–5.34)	<0.001	0.66 (0.56–0.78)
Physically inactive AF	1274	9.72 (9.19–10.26)		Reference
**All-cause death**				
Physically active AF	125	2.33 (1.94–2.78)	<0.001	0.52 (0.42–0.65)
Physically inactive AF	868	6.29 (5.88–6.73)		Reference
**MACE**				
Physically active AF	160	3.12 (2.65–3.64)	<0.001	0.80 (0.65–0.99)
Physically inactive AF	793	6.02 (5.60–6.45)		Reference
**CV death**				
Physically active AF	41	0.77 (0.55–1.04)	<0.001	0.60 (0.42–0.86)
Physically inactive AF	331	2.40 (2.15–2.67)		Reference
**Acute Coronary Syndrome**				
Physically active AF	77	1.49 (1.18–1.86)	<0.001	0.83 (0.59–1.16)
Physically inactive AF	341	2.56 (2.30–2.85)		Reference
**Thromboembolic Events**				
Physically active AF	52	1.03 (0.77–1.35)	0.078	1.03 (0.72–1.46)
Physically inactive AF	178	1.36 (1.16–1.57)		Reference
**Major Bleeding**				
Physically active AF	62	1.19 (0.91–1.53)	0.663	1.12 (0.80–1.56)
Physically inactive AF	173	1.28 (1.10–1.48)		Reference

**Figure 1:**
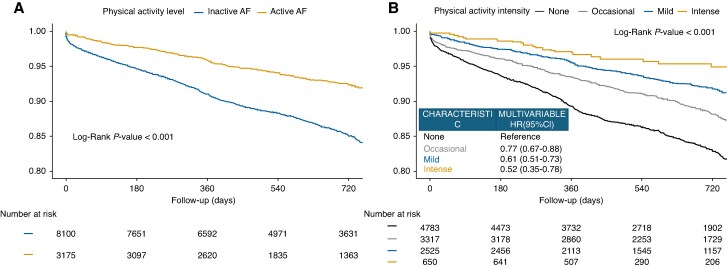
Kaplan–Meier curves for the risk of composite outcome in patients with atrial fibrillation *physically active* and *physically inactive* (*A*). Kaplan–Meier curves and Hazard Ratios (HR) with 95% Confidence Intervals (CI) for the risk of composite outcome in patients with different physically active intensity: *none* (no exercise or exercise for <3 h/week for < 2 years), *occasional* (<3 h/week), *mild* (≥3 h/week), *intense* (>7 h/week) (*B*). Adjusted for: age ≥ 75, sex, study group (European vs. Asian), obesity, symptomatic AF, hypertension, coronary artery disease (CAD), peripheral vascular disease (PAD), heart failure, diabetes mellitus, chronic kidney disease (CKD), dementia, history of thromboembolic events, anaemia and use of oral anticoagulants (OACs).

Univariable Cox regression analysis showed that, compared to *physically inactive* AF patients, *physically active* AF patients had a lower risk of composite outcome (HR 0.48, 95%CI 0.41–0.55, *Figure 1A*), all-cause death (HR 0.37, 95%CI 0.31–0.45), MACE (HR 0.54, 95%CI 0.44–0.65), cardiovascular death (HR 0.32, 95%CI 0.23–0.44), acute coronary syndrome (HR 0.61, 95%CI 0.44–0.83). A trend towards a reduced risk of thromboembolic events was observed, although it was not statistically significant (HR 0.80, 95%CI 0.58–1.09), while no significant difference was found for major bleeding (HR 0.93, 95%CI 0.69–1.24).

Multivariable Cox regression analysis (*Table 3*) confirmed that *physically active* AF was independently associated with a lower risk of composite outcome (HR 0.66, 95%CI 0.56–0.78), all-cause death (HR 0.52, 95%CI 0.42–0.65), MACE (HR 0.80, 95%CI 0.65–0.99), cardiovascular death (HR 0.60, 95%CI 0.42–0.86). No significant differences were observed for acute coronary syndrome, thromboembolic events, and major bleeding. Assessment of Schoenfeld residuals showed that the proportional hazards assumption was satisfied for physical activity (*P* = 0.787), although the global test (*χ*² = 37.45, *P* = 0.002) indicated some departure from proportionality driven by selected covariates (see Supplementary material online, *Table S5*).

When comparing *European* and *Asian* patients, no statistically significant interactions were observed for the risk of the composite outcome (*p*_interaction_ = 0.298), all-cause death (*p*_interaction_ = 0.340), MACE (*p*_interaction_ = 0.327), cardiovascular death (*p*_interaction_ = 0.530); however, the reductions in MACE (HR 0.60, 95%CI 0.32–1.12 vs. HR 0.83, 95%CI 0.67–1.04) and cardiovascular death (HR 0.41, 95%CI 0.12–1.44 vs. HR 0.63, 95%CI 0.43–0.90) appeared stronger in European patients than in their Asian counterparts (see Supplementary material online, *Table S6*).

### Risk of primary outcome across different physical activity levels

A progressive reduced risk of composite outcome was found in AF patients with moderate and intense physical activity compared to those with no physical activity (*Figure 1B*). These findings were also confirmed after adjustment for potential confounders. Compared to *no physically active* AF, *intense physically active* AF had the lowest risk of the composite outcome (HR 0.52, 95%CI 0.35–0.78), followed by *mild physically active* AF (HR 0.61, 95%CI 0.51–0.73), and *occasional physically active* AF (HR 0.77, 95%CI 0.67–0.88, *Figure 1B*). The results were consistent between *European* and *Asian* patients (*p*_interaction_ = 0.845); however, the association for occasionally physically active patients in the Asian subgroup was less pronounced (HR 0.80, 95%CI 0.46–1.40, Supplementary material online, *Table S7*).

### Risk of primary outcome after propensity score matching

A total of 3207 *physically active* AF patients (28.5% aged ≥75 years, 28.3% female) and 7942 *physically inactive* AF patients (36.1% aged ≥75 years, 41.9% female) who had complete data for all variables were included in the PSM (see Supplementary material online, *Table S8*).

**Figure 2:**
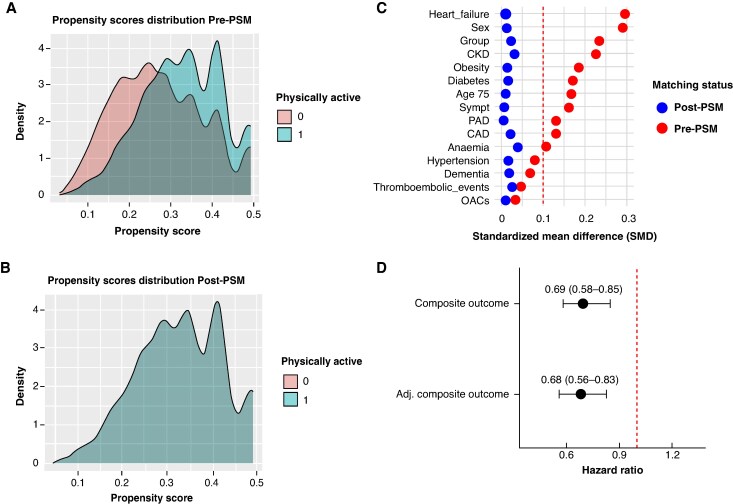
Distribution of propensity scores among *physically active* and *physically inactive* patients with atrial fibrillation before (*A*) and after (*B*) propensity score matching, with corresponding changes in absolute standardized mean differences (*C*). Forest plot showing hazard ratios and 95% confidence intervals from univariable and multivariable Cox regression analyses for the association between physical activity status (*physically active* vs. *physically inactive*) and the risk of the composite outcome after propensity score matching (panel D).

Before matching, *physically active* AF patients had a lower prevalence of cardiovascular burden, symptomatic AF, CKD, anaemia, dementia, and thromboembolic events, and were less frequently aged ≥75 years or female, compared with *physically inactive* AF patients (see Supplementary material online, *Table S8*). After PSM, 3207 patients were included in each group, and no differences were found between 2 groups (*Figure 2*, Supplementary material online, *Table S8*).

On multivariable Cox regression analysis after PSM, *physically active* AF patients were independently associated with a reduced risk of the composite outcome (HR 0.68, 95% CI 0.56–0.83, *Figure 2*). No violation of the proportional hazard assumption was detected based on Schoenfeld residuals (*χ^2^* = 20.3, *P* for proportionality =0.205).

### Robustness to unmeasured confounding (*E*-values)

The association between *physically active* patients and a lower risk of the composite outcome showed moderate robustness to unmeasured confounding. In the primary adjusted Cox model (HR 0.66, 95% CI 0.56–0.78), the *E*-value was 2.38 for the point estimate and 1.87 for the confidence interval limit closest to the null. In the propensity score–matched analysis (HR 0.68, 95% CI 0.56–0.83), the corresponding *E*-values were 2.30 and 1.72, respectively.

### Adjustment for anticoagulant type

After adjustment for OACs type, *physically active* patients remained independently associated with a lower risk of the composite outcome (HR 0.67, 95% CI 0.57–0.79), with effect estimates comparable to those observed in the main analysis.

### Subgroup analysis

**Figure 3:**
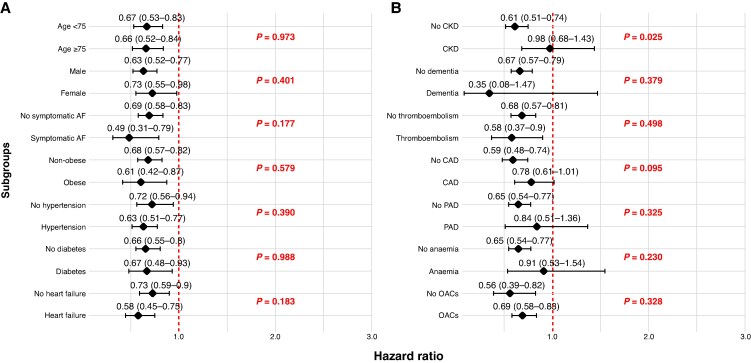
Risk of composite outcome in patients *physically active* with atrial fibrillation according to different subgroups. Hazard ratios (HRs) and 95% confidence intervals (CIs) are shown for each subgroup. Values of *P* for interaction in red.

A more pronounced beneficial effect of physical activity was found in AF patients without CKD compared to those with CKD (*P* for interaction = 0.024). No other statistically significant interactions were identified across subgroups (*Figure 3*).

### Competing risk analysis

In competing risk analyses accounting for non-cardiovascular death, *physically active AF* remained associated with a lower risk of cardiovascular death (sHR 0.62, 95% CI 0.43–0.88) and MACE (sHR 0.80, 95% CI 0.65–0.99).

## Discussion

In this study, our main findings are as follows: (i) *physically active* AF patients were younger, more often from Asia-Pacific registry, male, and had a lower prevalence of cardiovascular comorbidities compared to *physically inactive* AF patients; (ii) among both physically active and physically inactive groups, *Asian* patients were less likely to be obese and had lower odds of symptomatic AF and heart failure, but higher odds of cardiovascular risk factors such as hypertension and diabetes compared to *European;* (iii) *physically active* AF patients had a lower risk of the composite outcome, all-cause death, cardiovascular death, and MACE, with the risk decreasing progressively with increasing levels of physical activity; (iv) the reduced risk of the composite outcome, all-cause death, cardiovascular death, and MACE among physically active AF was consistent across both *Europeans* and *Asians*; (v) subgroup analyses revealed that physical activity was associated with a greater benefit in patients without CKD compared to those with CKD.

The baseline characteristics observed in *physically active* AF patients in our study are consistent with previous population-based cohort studies,^22–24^ which reported that *physically active* AF individuals were predominantly younger males with a lower cardiovascular burden. Gender differences in physical activity are well established^25,26^ and tend to be more pronounced in countries with lower levels of gender equality.^27^ Indeed, nations with greater gender inequality are less likely to provide public childcare or high-paying employment opportunities for women, potentially creating both time and financial barriers to their participation in physical activity.^28,29^

Physical inactivity can lead to weight gain, and studies have shown that adults with obesity engage in significantly less physical activity than their healthy-weight counterparts, creating a vicious cycle.^30^ These findings underscore the bidirectional relationship between physical inactivity and obesity whereby insufficient physical activity not only promote excessive weight gain but may also result from it.

### Comparing characteristics of European vs. Asian patients with AF

Asian origin was independently associated with a higher likelihood of being physically active, even after adjustment for potential confounders. This may reflect differences in baseline characteristics between the two enrolment settings. For instance, the higher prevalence of both symptomatic AF and heart failure among European individuals may contribute to reduced exercise tolerance, which in turn could lead to lower participation in physical activity.^11,31^ In this contest, our finding appears to contrast with a global analysis of 507 surveys from 163 countries and territories (2000–2022), which reported a higher prevalence of physical inactivity (36.5%) in the high-income Asia Pacific region compared to high-income Western countries (30.1%) and Central and Eastern Europe (18.0%).^2^ This discrepancy is likely explained, at least in part, by the clinic-based nature of the present registries, which enrolled patients attending cardiology care rather than individuals from the general population. Patients included in specialized AF registries may represent a more health-aware subgroup, with better access to healthcare and greater engagement in lifestyle modifications, including physical activity. These findings highlight the need for larger cohort studies specifically designed to assess the prevalence and patterns of physical activity in patients with AF. Such investigations are particularly important in light of the beneficial effects on the risk of adverse events observed in this study.

### Impact on outcomes

The lower risk of cardiovascular morbidity and mortality observed among *physically active* AF patients is consistent with findings from a previous prospective study conducted by Garnvik *et al*. in Norway.^23^ In a cohort of 1117 individuals with AF followed from 2006–2008 to 2015, they reported that those meeting general physical activity recommendations^32^ had a 45% lower risk of all-cause mortality compared with inactive individuals. Moreover, adherence to recommended physical activity levels was associated with a 22% lower risk of cardiovascular death. In a retrospective cohort study using data from the Korea National Health Insurance Service, Ahn et al.^24^ similarly showed that performing exercise any time before or after AF diagnosis was related to a lower risk of mortality and heart failure compared to those without exercise.

Given the substantial baseline differences between *physically active* and *physically inactive* patients, the presence of healthy participant bias is plausible. In this context, the lower CHA₂DS₂-VASc scores and the lower prevalence of symptomatic AF observed among *physically active* patients suggest that *physically inactive* patients may have been less active due to greater frailty, higher symptom burden, or more advanced comorbidity, raising the possibility of reverse causality. However, the consistency of the association after propensity score matching, together with the magnitude of the *E*-values, suggests that healthy participant bias alone is unlikely to fully account for the observed association, although residual confounding remains possible.

It is also important to consider that higher physical activity may act as a marker of better health status, which is independently associated with improved outcomes. Indeed, healthy lifestyle behaviours tend to cluster with each other, and individuals engaging in regular physical activity are often more health-conscious and more engaged in their care, which is associated with a lower risk of incident AF,^33^ and AF-related complications.^24,34,35^ In line with this concept, *physically active* patients showed a higher prevalence of adherence to the holistic ABC pathway.

In our analysis, we found no significant differences in the benefits of exercise between European and Asian patients with AF. This expands upon previous studies by providing new comparative evidence that the protective effects of physical activity on cardiovascular outcomes and mortality are similarly observed across distinct geographical populations. This finding highlights the generalizability of the benefits of physical activity, irrespective of geographical or ethnic background. This aspect is particularly relevant given the substantial clinical differences we observed between European and Asian patients in terms of cardiometabolic profiles. Indeed, in other clinical contexts, such ethnic differences have been shown to significantly influence treatment response. For example, studies have reported that the risk reduction in major bleeding associated with non–vitamin K antagonist oral anticoagulants (NOACs) is greater in Asian populations compared with non-Asians, while Asians have been shown to be at higher risk of intracranial haemorrhage.^36^

The possible mechanisms linking physical activity to improved cardiovascular outcomes in patients with AF are varied and not yet completely understood. Some of these mechanisms may not be directly related to AF itself, but rather to common comorbidities that frequently coexist with AF, such as hypertension, diabetes, and atherosclerosis. Indeed, physically active individuals tend to exhibit lower blood pressure, enhanced insulin sensitivity, and a more favourable plasma lipoprotein profile, all of which may contribute to improved prognosis.^37^ Moreover, physical activity may also exert direct effects on the heart by promoting cellular changes in cardiomyocytes. In this context, it not only induces beneficial metabolic and molecular remodelling, but also increases levels of circulating progenitor cells, which have been implicated in supporting cardiac repair^38^ and improves cardiac vascularization, providing protection against vascular stress and reducing the risk of cardiac events.^39^ Finally, exercise has been shown to lower systemic inflammation, which is typically elevated in cardiovascular disease and obesity.^40^

However, despite the well-established benefits of physical activity, extreme endurance exercise has been associated with adverse structural changes in the heart, including the progression of coronary artery calcification,^41^ myocardial fibrosis,^42^ and pathological cardiac remodelling, including enlargement of all four heart chambers.^43^ In particular, the combination of left atrial enlargement and increased parasympathetic tone linked to sustained physical activity may may increase the risk of developing AF.^43^ Nevertheless, there is limited evidence on the impact of high-intensity exercise in patients with established AF. Accordingly, current guidelines recommend moderate- to vigorous-intensity aerobic exercise, targeting 210 min per week.^44^ In this context, our results showed a progressively lower risk of adverse outcomes with increasing levels of physical activity. Our results are consistent with those of Garnvik *et al*.^23^ who reported that the risk of each outcome was slightly lower among individuals engaging in vigorous-intensity activity compared to moderate-intensity, across equivalent weekly durations of exercise.

Lastly, we found a lower benefit of physical activity was observed in patients with CKD compared to those without CKD. This may be partially explained by the multiple comorbidities commonly associated with CKD. Indeed, conditions such as hypertension, dyslipidaemia, obesity, and diabetes independently contribute to an elevated risk of cardiovascular events and require more complex therapeutic management.^45–47^ In addition, patients with CKD frequently present features of frailty, including reduced exercise tolerance, sarcopenia, anaemia, and functional limitations, which may restrict both the intensity and sustainability of physical activity, thereby attenuating its potential cardiovascular benefits.^48^ Furthermore, CKD represents a heterogeneous group of disorders with varying severity and underlying pathophysiology, which were not differentiated in our analysis.

The rising levels of physical inactivity worldwide, particularly in regions undergoing rapid urbanization,^49^ underscore the importance of physical activity as a key non-pharmacological treatment strategy for patients with AF. Greater efforts are therefore needed to promote its adoption in clinical care. Although physical activity is formally included in the holistic or integrated care approach proposed by the ABC pathway.^50^ The latter is supported by evidence from clinical trials and large cohort studies,^51–53^ and is recommended in contemporary management pathways and guidelines.^54,55^ Nevertheless, the role of exercise is often underestimated by patients^56^ and not considered a priority by healthcare providers.^57^

## Limitations

This was a post-hoc analysis of an observational study, so caution is needed when inferring causal relationships between physical activity and cardiovascular outcomes in patients with AF, as the data only suggest potential associations rather than definitive cause-and-effect conclusions. Second, physical activity was assessed once at baseline using self-reported information and not through a validated questionnaire or device-based measurement, which may be subject to recall and reporting bias. This approach did not allow evaluation of changes in physical activity over time, nor of the potential impact of AF progression or symptom worsening during follow-up. Accordingly, exposure misclassification and temporal ambiguity cannot be excluded. In addition, reverse causality remains possible, as patients with more advanced disease, greater symptom burden, or reduced functional capacity may have been less likely to engage in physical activity at baseline. Third, physical activity was classified based on self-reported hours per week and did not allow MET-based or device-derived quantification of exercise intensity, which would provide more accurate estimates of its intensity. Accordingly, intensity was explored indirectly through predefined activity categories rather than measured directly. Fourth, the two registries differed in follow-up duration, enrolment period, and healthcare context, and although time-to-event analyses were used, such structural heterogeneity cannot be fully accounted for analytically and should be considered when interpreting the results. Fifth, the models were adjusted for potential confounders; there may be other confounding factors not considered that could influence the results. Sixth, patients were divided according to the site of enrolment (Europe and Asia) rather than ethnicity. As such, the lack of an ethnic-based classification could affect the interpretation of regional variations and the generalizability of the findings across specific ethnic groups, especially given the reported ethnic differences in clinical epidemiology and AF-related outcomes (e.g. stroke, bleeding).^58–60^ Finally, differences in the severity of CKD were not considered, which may have affected the assessment of physical activity benefits in this group.

## Conclusion

In this large, multinational cohort of patients with AF, self-reported physical activity was associated with a lower risk of adverse events, with a consistent dose–response relationship. The reduction in risk was consistent across Europe and Asia. Physical activity may therefore identify a lower-risk phenotype among patients with AF, although further research is needed to clarify its potential role in risk modification.

## Supplementary Material

euag032_Supplementary_Data

## Data Availability

Data used in this research project will be available upon a reasonable request from the corresponding authors.
