# Supplementary material for: Physical activity and risk of adverse events in atrial fibrillation: evidence from European and Asian cohorts
Source: Europace. 2026 Mar 1;28(2):euag032. doi: 10.1093/europace/euag032 (PMC12964361; doi:10.1093/europace/euag032)

**Physical Activity and Risk of Adverse Events in Atrial Fibrillation: Evidence from European and Asian Cohorts**

Michele Rossi, Tommaso Bucci, Enrico Tartaglia, Amir Askarinejad,

Steven Ho Man Lam, Andrea Galeazzo Rigutini, Claudio Ferri, Giuseppe Boriani,

Hung-Fat Tse, Tze-Fan Chao, Gregory Y. H. Lip

Supplementary material

**Supplementary Table 1.** Univariable and Multivariable Logistic Regression analysis of factors associated with *physical activity* in patients with atrial fibrillation.

| **CHARACTERISTIC** | **UNIVARIABLE**  OR (95% CI) | **MULTIVARIABLE**  OR (95% CI) |
| --- | --- | --- |
| Age ≥ 75 | 0.69 (0.64-0.73) | 0.84 (0.77-0.93) |
| Female | 0.57 (0.52-0.62) | 0.57 (0.52-0.63) |
| Asian | 1.73 (1.59-1.87) | 1.41 (1.28-1.55) |
| Obesity | 0.67 (0.60-0.73) | 0.74 (0.67-0.82) |
| Symptomatic AF | 0.60 (0.54-0.68) | 0.78 (0.67-0.89) |
| Hypertension | 0.85 (0.78-0.92) | 1.00 (0.92-1.10) |
| Diabetes | 0.66 (0.60-0.73) | 0.78 (0.70-0.87) |
| Heart failure | 0.53 (0.49-0.58) | 0.65 (0.59-0.72) |
| CKD | 0.46 (0.40-0.53) | 0.59 (0.50-0.70) |
| Dementia | 0.48 (0.32-0.68) | 0.75 (0.47-1.16) |
| Thromboembolic events | 0.84 (0.74-0.95) | 0.95 (0.83-1.10) |
| CAD | 0.73 (0.66-0.80) | 0.89 (0.81-1.00) |
| PAD | 0.56 (0.46-0.67) | 0.80 (0.65-0.98) |
| Anaemia | 0.62 (0.52-0.74) | 0.85 (0.69-1.04) |
| OACs | 1.00 (0.90-1.11) | 1.00 (0.89-1.13) |

Adjusted for: age≥ 75, sex, study group (European vs. Asian), obesity, symptomatic AF, hypertension, coronary artery disease (CAD), peripheral vascular disease (PAD), heart failure, diabetes mellitus, chronic kidney disease (CKD), dementia, history of thromboembolic events, anaemia and use of oral anticoagulants (OACs).

**Supplementary Table 2**. Baseline characteristics of *European* and *Asian* physically active patients with atrial fibrillation. *BMI* body mass index, *CKD* chronic kidney disease*, CAD* coronary artery disease*, PAD* peripheral vascular disease, *OACs* oral anti-coagulants.

| **Characteristic** | **European** N = 2,342 | **Asian** N = 1,297 | **p-value** |
| --- | --- | --- | --- |
| Age | 66 ± 12 | 71 ± 11 | <0.001 |
| Sex |  |  | 0.050 |
| Male | 1,669 (71%) | 884 (68%) |  |
| Female | 673 (29%) | 413 (32%) |  |
| Smoke | 240 (10%) | 89 (7.0%) | <0.001 |
| Obesity | 587 (26%) | 117 (9.9%) | <0.001 |
| Symptomatic AF | 357 (15%) | 58 (4.5%) | <0.001 |
| Hypertension | 1,290 (55%) | 863 (67%) | <0.001 |
| Diabetes | 354 (15%) | 324 (25%) | <0.001 |
| Dyslipidaemia | 834 (37%) | 538 (42%) | 0.012 |
| Heart failure | 652 (28%) | 291 (23%) | <0.001 |
| Dementia | 13 (0.6%) | 20 (1.5%) | 0.003 |
| CAD | 528 (23%) | 270 (21%) | 0.11 |
| PAD | 131 (5.7) | 17 (1.3%) | <0.001 |
| CKD | 151 (6.5%) | 90 (6.9%) | 0.6 |
| Cancer | 38 (1.6%) | 37 (2.9%) | 0.013 |
| Thromboembolic events | 217 (9.3%) | 165 (13%) | <0.001 |
| Anaemia | 51 (2.2%) | 113 (8.7%) | <0.001 |
| CHA_2_DS_2_VASc | 2.48 ± 1.64 | 2.92 ± 1.64 | <0.001 |
| CHA_2_DS_2_VA | 2.19 ± 1.56 | 2.60 ± 1.53 | <0.001 |
| HASBLED | 1.37 ± 1.03 | 1.61 ± 1.03 | <0.001 |
| OACs | 1,972 (84%) | 1,075 (83%) | 0.3 |

**Supplementary Table 3**. Baseline characteristics of *European* and *Asian* physically inactive patients with atrial fibrillation. *BMI* body mass index, *CKD* chronic kidney disease*, CAD* coronary artery disease*, PAD* peripheral vascular disease, *OACs* oral anti-coagulants.

| **Characteristic** | **European** N = 7,183 | **Asian** N = 2,304 | **p-value** |
| --- | --- | --- | --- |
| Age | 70 ± 11 | 68 ± 12 | <0.001 |
| Sex |  |  | <0.001 |
| Male | 3,952 (55%) | 1,471 (64%) |  |
| Female | 3,231 (45%) | 833 (36%) |  |
| Smoke | 639 (9.2%) | 196 (8.7%) | 0.4 |
| Obesity | 2,199 (33%) | 251 (12%) | <0.001 |
| Symptomatic AF | 1,495 (21%) | 174 (7.6%) | <0.001 |
| Hypertension | 4,566 (64%) | 1,399 (61%) | 0.008 |
| Diabetes | 1,853 (26%) | 579 (25%) | 0.6 |
| Dyslipidaemia | 2,960 (43%) | 897 (39%) | 0.002 |
| Heart failure | 3,247 (46%) | 520 (23%) | <0.001 |
| Dementia | 130 (1.8%) | 48 (2.1%) | 0.4 |
| CAD | 2,083 (31%) | 484 (21%) | <0.001 |
| PAD | 644 (9.1%) | 24 (1.0%) | <0.001 |
| CKD | 1,056 (15%) | 198 (8.6%) | <0.001 |
| Cancer | 147 (2.1%) | 53 (2.3%) | 0.5 |
| Thromboembolic events | 879 (12%) | 280 (12%) | >0.9 |
| Anaemia | 496 (6.9%) | 174 (7.6%) | 0.3 |
| CHA_2_DS_2_VASc | 3.39 ± 1.77 | 2.74 ± 1.77 | <0.001 |
| CHA_2_DS_2_VA | 2.94 ± 1.65 | 2.38 ± 1.65 | <0.001 |
| HASBLED | 1.66 ± 1.10 | 1.35 ± 1.08 | <0.001 |
| OACs | 6,091 (85%) | 1,859 (81%) | <0.001 |

**Supplementary Table 4**. Baseline characteristics of missing data group for follow-up

| **Characteristic** | **Group**  **included** N = 11,275 | **Missing**  **data group**  N = 1,851 | p-value |
| --- | --- | --- | --- |
| Age | 69 ± 12 | 69 ± 12 | 0.041 |
| Sex |  |  | >0.9 |
| Male | 6,850 (61%) | 1,126 (61%) | >0.9 |
| Female | 4,425 (39%) | 725 (39%) | >0.9 |
| Group |  |  | <0.001 |
| European | 7,722 (73%) | 1,198 (68%) |  |
| Asian | 2,912 (27%) | 555 (32%) |  |
| Smoke | 950 (8.7%) | 214 (12%) | <0.001 |
| Obesity | 2,799 (27%) | 355 (21%) | <0.001 |
| Hypertension | 6,946 (62%) | 1,172 (64%) | 0.2 |
| Diabetes | 2,655 (24%) | 455 (25%) | 0.3 |
| Dyslipidaemia | 4,497 (41%) | 732 (41%) | 0.8 |
| Heart failure | 4,026 (36%) | 684 (37%) | 0.3 |
| Dementia | 184 (1.6%) | 27 (1.5%) | 0.6 |
| CAD | 2,765 (26%) | 600 (35%) | <0.001 |
| PAD | 696 (6.3%) | 120 (6.6%) | 0.5 |
| CKD | 1,270 (11%) | 225 (12%) | 0.3 |
| Cancer | 233 (2.1%) | 42 (2.3%) | 0.6 |
| Thromboembolic events | 1,336 (12%) | 205 (11%) | 0.3 |
| Ischaemic stroke | 743 (6.6%) | 125 (6.8%) | >0.9 |
| Anaemia | 710 (6.3%) | 124 (6.7%) | 0.5 |
| CHA_2_DS_2_VASc | 3.06 ± 1.77 | 3.09 ± 1.79 | 0.5 |
| CHA_2_DS_2_VA | 2.67 ± 1.65 | 2.70 ± 1.66 | 0.4 |
| HASBLED | 1.54 ± 1.08 | 1.59 ± 1.11 | 0.081 |
| OACs | 9,573 (85%) | 1,424 (77%) | <0.001 |

**Supplementary Table 5**. Schoenfeld residual test p-values for each covariate in the Cox model for the composite outcome.

| **Covariate** | **χ²** | **df** | **p-value** |
| --- | --- | --- | --- |
| Physical activity | 0.07 | 1 | 0.787 |
| Age ≥75 years | 4.60 | 1 | 0.032 |
| Sex | 1.26 | 1 | 0.261 |
| Group | 12.48 | 1 | <0.001 |
| Obesity | 0.26 | 1 | 0.613 |
| Hypertension | 0.16 | 1 | 0.693 |
| Diabetes | 0.01 | 1 | 0.932 |
| Symptoms | 0.72 | 1 | 0.395 |
| Heart failure | 6.27 | 1 | 0.012 |
| CKD | 0.08 | 1 | 0.772 |
| Dementia | 0.06 | 1 | 0.812 |
| Thromboembolic events | 0.19 | 1 | 0.664 |
| CAD | 1.13 | 1 | 0.287 |
| PAD | 3.87 | 1 | 0.049 |
| Anaemia | 0.45 | 1 | 0.501 |
| OACs | 2.80 | 1 | 0.095 |
| **Global test** | **37.45** | **16** | **0.002** |

**Supplementary Table 6**. Risk of outcomes in *physically active* patients with atrial fibrillation according to different enrolment settings (HR: hazard ratio; CI: confidence interval). *Physically inactive* patients are the reference group.

| **Outcome** | **European**  HR (95%CI) | **Asian**  HR (95%CI) | **P interaction** |
| --- | --- | --- | --- |
| Composite outcome | 0.69 (0.58­­­­-­­­0.82) | 0.53 (0.34-0.83) | 0.298 |
| All-cause death | 0.55 (0.44-0.69) | 0.41 (0.23-0.72) | 0.340 |
| MACE | 0.83 (0.67-1.04) | 0.60 (0.32-1.12) | 0.327 |
| CV death | 0.63 (0.43-0.90) | 0.41 (0.12-1.44) | 0.530 |
| Acute Coronary Syndrome | 0.85 (0.60-1.21) | 0.65 (0.23-1.83) | 0.631 |
| Thromboembolic Events | 1.10 (0.76-1.61) | 0.64 (0.23-1.77) | 0.320 |
| Major Bleeding | 1.08 (0.75-1.57) | 1.29 (0.60-2.76) | 0.686 |

Adjusted for: age≥ 75, sex, study group (European vs. Asian), obesity, symptomatic AF, hypertension, coronary artery disease (CAD), peripheral vascular disease (PAD), heart failure, diabetes mellitus, chronic kidney disease (CKD), dementia, history of thromboembolic events, anaemia and use of oral anticoagulants (OACs).

**Supplementary Table 7**. Risk of primary outcome across different physical activity levels in according to different enrolment settings (HR: hazard ratio; CI: confidence interval). *No physically active* patients are the reference group.

| **Physical activity levels** | **European**  HR (95%CI) | **Asian**  HR (95%CI) | **P interaction** |
| --- | --- | --- | --- |
| Occasional physically active | 0.74 (0.62-0.87) | 0.80 (0.46-1.40) | 0.845 |
| Mild physically active | 0.48 (0.37-0.62) | 0.39 (0.20-0.76) | 0.845 |
| Intense physically active | 0.49 (0.27-0.88) | 0.35 (0.13-0.97) | 0.845 |

Adjusted for: age≥ 75, sex, study group (European vs. Asian), obesity, symptomatic AF, hypertension, coronary artery disease (CAD), peripheral vascular disease (PAD), heart failure, diabetes mellitus, chronic kidney disease (CKD), dementia, history of thromboembolic events, anaemia and use of oral anticoagulants (OACs).

**Supplementary Table 8**. Baseline characteristics of *physically active* and *physically inactive* patients with AF before and after Propensity Score Matching (PSM).

|  | **Before PSM** | | | **After PSM** | | |
| --- | --- | --- | --- | --- | --- | --- |
|  | **Physically active**  **N= 3,207** | **Physically inactive**  **N= 7,942** | **SMD** | **Physically active**  **N= 3,207** | **Physically inactive**  **N= 3,207** | **SMD** |
| Age ≥75 years, n (%) | 913 (28.5) | 2871 (36.1) | 0.165 | 913 (28.5) | 901 (28.1) | 0.008 |
| Female sex, n (%) | 906 (28.3) | 3330 (41.9) | 0.290 | 906 (28.3) | 921 (28.7) | 0.010 |
| Group = 1, n (%) | 1125 (35.1) | 1941 (24.4) | 0.234 | 1125 (35.1) | 1094 (34.1) | 0.020 |
| Obesity, n (%) | 645 (20.1) | 2217 (27.9) | 0.183 | 645 (20.1) | 660 (20.6) | 0.012 |
| Hypertension, n (%) | 1887 (58.8) | 4974 (62.6) | 0.078 | 1887 (58.8) | 1865 (58.2) | 0.014 |
| Diabetes, n (%) | 595 (18.6) | 2027 (25.5) | 0.169 | 595 (18.6) | 579 (18.1) | 0.013 |
| Symptoms, n (%) | 354 (11.0) | 1313 (16.5) | 0.160 | 354 (11.0) | 351 (10.9) | 0.003 |
| Heart failure, n (%) | 783 (24.4) | 3006 (37.8) | 0.293 | 783 (24.4) | 794 (24.8) | 0.008 |
| CKD, n (%) | 201 (6.3) | 1019 (12.8) | 0.225 | 201 (6.3) | 180 (5.6) | 0.028 |
| Dementia, n (%) | 25 (0.8) | 116 (1.5) | 0.065 | 25 (0.8) | 21 (0.7) | 0.015 |
| Thromboembolic events, n (%) | 338 (10.5) | 945 (11.9) | 0.043 | 338 (10.5) | 316 (9.9) | 0.023 |
| CAD, n (%) | 718 (22.4) | 2217 (27.9) | 0.128 | 718 (22.4) | 693 (21.6) | 0.019 |
| PAD, n (%) | 130 (4.1) | 558 (7.0) | 0.130 | 130 (4.1) | 129 (4.0) | 0.002 |
| Anaemia, n (%) | 130 (4.1) | 508 (6.4) | 0.105 | 130 (4.1) | 108 (3.4) | 0.036 |
| OACs, n (%) | 2682 (83.6) | 6729 (84.7) | 0.030 | 2682 (83.6) | 2692 (83.9) | 0.008 |

**Supplementary Figure 1**. Study flowchart


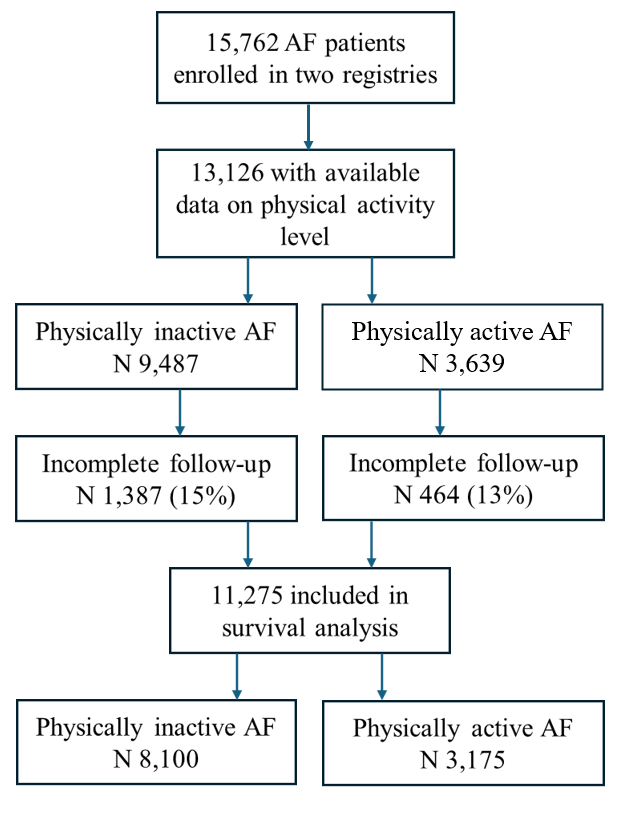

Supplement: euag032_Supplementary_Data [file euag032_supplementary_data.docx]
